# Feasibility of a preoperative strengthening exercise program on postoperative function in patients undergoing hip or knee arthroplasty: a pilot randomized controlled trial

**DOI:** 10.1186/s40814-022-01126-9

**Published:** 2022-07-30

**Authors:** Lissa Pacheco-Brousseau, Johanna Dobransky, Alanna Jane, Paul E. Beaulé, Stéphane Poitras

**Affiliations:** 1 Faculty of Health Sciences, School of Rehabilitation Sciences, University of Ottawa, 451 Smyth Road, Ottawa, Ontario K1H 8M5 Canada; 2 Division of Orthopaedic Surgery, The Ottawa Hospital, Ottawa, Ontario Canada; 3 Faculty of Medicine, Department of Surgery, University of Ottawa, Ottawa, Ontario Canada

**Keywords:** Hip arthroplasty, Knee arthroplasty, Joint arthroplasty, Preoperative exercises, Osteoarthritis, Randomized control trial, Feasibility study, Pilot study

## Abstract

**Background:**

There are conflicting results on the effect of preoperative exercise programs on long-term function and little evidence on short-term function. The aim is to assess the feasibility of a preoperative strengthening exercise program in patients undergoing hip or knee joint arthroplasty in terms of trial design, recruitment, and follow-up rates.

**Methods:**

A randomized controlled feasibility study with patients undergoing hip or knee joint arthroplasty. Patients were randomized to a preoperative strengthening exercise program or standard of care. Feasibility outcome measures were recruitment rate (≥ 50%) and loss to follow-up (≤ 15%).

**Results:**

Of the 129 eligible participants, 63 participants consented to participate in the study (49%), and 27 were successfully randomized prior to surgery (43%). All 27 participants completed the baseline assessment. Of these, 6 (22%) had surgery during the exercise period. Of the remaining 21 participants, 20 (95%) completed the pre-surgery assessment. The study was terminated before five participants could be eligible for the 6-month assessment. Sixteen (76%) participants completed the 6-week post-surgery assessment. Twelve participants completed the 6-month assessment (75%).

**Conclusion:**

Given the recruitment rate, randomization barriers, and study participant loss to follow-up, the study was discontinued since it was not considered feasible in this current form at our clinical site despite modifications made to the protocol. Future investigations into a modified intervention via telerehabilitation should be explored.

**Trial registration:**

ClinicalTrials.gov, NCT03483519. Retrospectively registered in March 2018.

## Key messages regarding feasibility

- The goal of this pilot study was to investigate the volume of eligible patients, patient acceptability rate, reasons for declining participation, feasibility of randomization and intervention, and outcome completion rates at all time periods.
- This pilot randomized controlled trial suggests that in-person preoperative strengthening exercise program is not optimal for patients undergoing hip and knee joint arthroplasty due to travel constraints and time requirements.
- Future studies should investigate the delivery of a preoperative strengthening exercise program for patients undergoing hip and knee joint arthroplasty surgery using telerehabilitation.

## Background

Joint arthroplasty is the indicated treatment to manage hip and knee pain and functional limitations due to a late-stage localized joint problem (osteoarthritis, avascular necrosis, etc.) (JA) [1, 2]. Hip and knee arthroplasties are commonly performed surgeries worldwide, and numbers are expected to continue rising due to increasing age and obesity rates in the general population [3–7]. In 2018, the number of hip and knee arthroplasty surgeries performed in Canada increased by 17.4% and 17.0%, respectively, compared to 5 years earlier [7]. Despite the success of JA, over half of patients spend four or more days in the hospital after surgery [7, 8], incurring substantial costs to the healthcare system. Improving quicker return to function after surgery to reduce hospital length of stay (LOS) and associated costs and allowing patients to be able to return to activity are a major focus of the healthcare system [9].

One of the key factors affecting recovery after JA is the patient’s preoperative physical function [10]. Thus, interventions addressing modifiable preoperative factors associated with postoperative function could be beneficial. Most patients undergoing JA demonstrate muscle deconditioning and decreased physical activity both before and after surgery [11–13], limiting their capacity to accomplish basic essential functional tasks and increasing the risk of falls [14]. Furthermore, many patients report persistent pain and functional disability and demonstrate reduced functional ability and return to work capacity over the term [15–17]. Although lower-limb musculature in general appears affected in patients undergoing JA [13, 18], hip abductors for hip osteoarthritis (OA) and quadriceps for knee OA have been shown to be the most affected muscle groups [19, 20]. Both are essential muscle groups when performing functional activities such as walking and stair climbing [21, 22].

Traditionally, exercises have been used postoperatively in the JA population [23, 24]. Due to the invasive nature of JA surgery, patient condition shortly after surgery is not optimal for accomplishing intensive exercises needed to address post-surgery deconditioning [23]. Furthermore, the patient is at increased risk of developing deep vein thrombosis and experiencing adverse events to the prosthesis [25–28]. The limited capacity to accomplish an activity in the recovery period can further decondition the muscles [25, 29]. Studies have demonstrated the potential benefits of exercises for patients with OA and patients having JA. A study assessing an 8-week home-based hip abductor strengthening program for patients with knee OA found significant improvements in hip abductor strength and decreased knee pain post-intervention [30]. Early preoperative progressive resistance exercise programs (beginning within a month prior to surgery) were also found to significantly improve muscle strength for patients undergoing hip JA [23]. Conversely, another systematic review [31] of a preoperative physiotherapy program showed improvements in hip muscle strength and WOMAC scores for patients having hip JA, but no significant improvements for knee JA. However, the potential benefits of preoperative exercise programs on postoperative function in JA remain unclear due to the poor therapeutic validity of available studies [17].

To address postoperative exercise shortcomings, preoperative exercises have been studied but have shown both positive and negative outcome results [17, 25, 31–33]. This may be attributable to the low methodology of included studies, with reviews acknowledging the need for stronger quality trials [32, 33]. Current studies comprised various limitations such as (1) exercise programs do not specifically target muscles associated with function and instead rely on generic exercise programs; (2) exercise parameters are often vague, which limits the capacity to apply the intervention; (3) most trials did not assess both short- and long-term function after hospitalization, which leads to limited evidence on the effect of preoperative exercises on short and long term benefits; and finally, (4) many trials used exercise programs that were not deemed feasible or valid for the JA population [17, 23, 25, 31].

Although specific muscle impairments have been identified, no study to our knowledge has evaluated the impact of managing these patients preoperatively with regard to short- and long-term functions. To determine whether a large-scale trial was feasible, we proposed a randomized controlled feasibility study addressing the previously mentioned limitations. The aim was to assess the feasibility of a preoperative strengthening exercise program in patients undergoing hip or knee arthroplasty in terms of trial design, recruitment, and follow-up rates.

## Methods

This study was reported based on the CONSORT reporting checklist for pilot and feasibility trials [34].

### Design and setting

A single-blind randomized controlled feasibility study was conducted to evaluate the feasibility and potential efficacy of a home-based preoperative strengthening exercise program compared to the standard of care in patients undergoing JA. The trial was conducted at a large tertiary care center. The trial was registered at ClinicalTrials.gov (NCT03483519), and ethics approval was received from the Institutional Review Board (20150684).

### Participants

Patients undergoing unilateral JA were recruited from the clinical practices of six orthopedic surgeons performing elective arthroplasty at a large tertiary care center. The inclusion criteria were being 18 years of age and older, WOMAC functional subscale of less than 66.5/100 [35, 36], and undergoing unilateral total hip or knee arthroplasty due to OA. The exclusion criteria were other previously diagnosed lower-limb problems limiting their capacity to accomplish the exercise program, joint revision, bilateral arthroplasty, same-day discharge, surgery in less than 10 weeks after recruitment, pregnancy or suspected pregnancy, unable or unwilling to commit to required study follow-ups, no fixed address, and cognitive impairment that may preclude questionnaire completion.

### Recruitment and randomization

The initial recruitment method relied on administrative assistants contacting the research coordinator when a potential patient was cleared for surgery. The study coordinator provided details about the trial to the patient and asked for consent to participate. Patients who agreed to take part in the study signed the consent form, and a baseline assessment was conducted at the same moment. When it was not possible to meet patients in person, the study coordinator contacted the patient over the phone after the consultation appointment to schedule the consent process and baseline assessment at a convenient time. Participants were randomized using an institutional online software to either the intervention group (preoperative strengthening exercise program) or control group (standard of care) by the research coordinator directly after baseline assessment. A blocked stratified randomization procedure (varying between 2 and 4) was used stratifying by joint, age (< 68, ≥ 68) [7], gender, and preoperative WOMAC physical function subscale score (< 40, ≥ 40) [35]. Study assessors were blinded to the participants’ group allocation, but physiotherapists were aware of allocation.

### Intervention

The intervention consisted of a home-based preoperative strengthening exercise program specific to the participant condition (hip or knee JA) under the supervision of a physiotherapist. The exercise program targeted a specific muscle: hip abductor for participants with hip OA and quadriceps for participants with knee OA [37–39]. Each week, the difficulty level of the exercise increased. Exercise parameters were 3 sets of 10 repetitions, with a 1-min break between sets, five times a week, for a total of 8 weeks (see the Table 2 in Appendix for hip and knee exercise program details) [40, 41]. The capacity of the participant to perform the exercise and to progress was assessed each week during a session with a physiotherapist. During the visit, the physiotherapist demonstrated the exercise to the participant and evaluated the participant’s capacity to perform the exercise. Capacity was determined in two ways: (1) participant is physically capable of performing the exercise and (2) participant exertion rating on the Borg Rating of Perceived Exertion (RPE) scale during the completion of the exercise (rating needs to be ≥ 5) [42]. These two conditions had to be met for the exercise program to be used during the week. If one or both of the conditions were not met, the participant continued to use the preceding week’s exercise. Participants in the intervention group started the strengthening exercise program between 8 and 10 weeks prior to the patient surgery date. Participants could reach their attending surgeon if they had any questions with regard to the exercise program.

### Control

The intervention and control groups received the standard of care utilized at a large tertiary care center for hip and knee arthroplasties. The standard of care consists of preoperative patient education with an accompanying booklet. Education included information on the surgery and recovery process, anesthesia approach, different surgery approaches, postoperative education, early mobilization and ambulation, postoperative analgesia, discharge planning, and standardized postoperative range of motion exercises.

### Outcomes

#### Feasibility

Primary outcomes were associated with study feasibility and included recruitment rate, loss to follow-up, and missing data [43, 44]. Primary outcomes informed the feasibility of a larger randomized control trial (RCT). The following criteria had to be met to be considered as feasible in our study clinical setting: (1) recruitment rate of ≥ 50% and (2) loss to follow-up ≤ 15% [34, 44, 45]. The recruitment rate was defined as the number of patients successfully recruited and randomized from those eligible. Loss to follow-up was defined as participants who were withdrawn, dropped out, or missed follow-up assessment. Power calculation was used to inform recruitment rate feasibility. The minimal clinically important difference (MCID) for the Timed-Up-and-Go (TUG), which was the primary outcome, is 1.4 s [46]. The standard deviation (SD) of TUG in this patient population is approximately 2.5 s [46]. With an alpha level of 0.05 and 80% power, a sample size of 100 patients (50 per group) would be required for a full RCT. In order to account for potential study attrition, a 15% oversample rate needs to be used. Therefore, a sample of 118 patients (59 per group) would be required for a full RCT. Feasibility outcomes were recorded over a total of 11 months between February 2017 and November 2018 with recruitment breaks to allow recruitment strategy adjustment.

#### Outcome measures

Outcomes included performance-based function, patient-reported function, muscle strength (quadriceps or hip abductor), and muscle mass (bone density, percentage of fat, percentage of lean body mass). Performance-based and patient-reported function was assessed during orthopedic consultations at baseline, after the completion of the exercise program, and 6 weeks and 6 months after surgery. Muscle strength and muscle mass were assessed at the same intervals, except at 6 weeks after surgery because of acuteness. Assessors were blinded to the study participant group.

Performance-based function was assessed with the TUG and the Timed Stair Test. TUG assesses the time that a patient takes to rise from a chair, walk 3 m, turn around, walk back to the chair, and sit down. It has been demonstrated to be reliable, valid [47], and responsive in patients undergoing JA [48]. Furthermore, it has been shown to be predictive of hospital LOS, short- and long-term function [48–50]. The TUG has been extensively used as an outcome measure in trials with patients undergoing JA [48]. The Timed Stair Test assesses the time that a patient takes to ascend and descend a flight of 10 stairs, while holding on to the handrail [51]. It is a recommended test for JA patients [52]. Trained members of the research team assisted participants by explaining and demonstrating the tests and timed each participant attempt. Participants were instructed to do the TUG test twice at each assessment visit, with the fastest time of the two being used, and the TST once per assessment.

Patient-reported function included the HOOS and KOOS questionnaires [53, 54] for disease-specific outcomes and the EQ-5D-5L questionnaires for general health status and quality of life [55]. They have been shown to be a valid, reliable, and responsive measure among patients undergoing JA and are widely used in this field [53–55]. Both questionnaires were given to patients in person.

Muscle strength was assessed with a hand-held dynamometer by a trained research assistant. For patients undergoing hip surgery, hip abductor strength was assessed with the patient in the supine position. With the patient lying on an examination bed, a research assistant used the dynamometer to directly apply force to the leg (in the frontal plane, lateral to the midline) and instructed the patient to resist the applied force as much as possible. The dynamometer measured the strength exerted by the patient’s muscles in kilograms. For patients undergoing knee surgery, quadriceps strength was assessed with the patient in a sitting position with the knee flexed at 90°. With the patient sitting on an examination bed, a research stabilized the dynamometer to the leg using a stabilization band and instructed the patient to push against the band as much as possible (sagittal plane). The dynamometer measured the strength exerted in kilograms. The average of three trials was used. These methods have been shown to be valid and reliable measures for assessing hip [56, 57] and quadriceps strength [58–60]. Absolute (affected side only) and relative (difference between non-affected and affected side) strength values were calculated.

Muscle mass (bone density and percentage of fat and lean body mass) was assessed using the dual-energy X-ray absorptiometry (DEXA) method. Patients were lying on an examination table while a low-intensity X-ray scanned the entire body for approximately 20 min.

Patient demographics were collected at baseline during recruitment by the study coordinator.

#### Data analysis

The analysis was conducted on an intention-to-treat basis. Feasibility outcomes were measured using descriptive statistics. The Mann-Whitney *U* test was used to compare the study groups at baseline. A level of significance of 0.05 was used to compare the characteristics between the intervention and control groups. Analyses were accomplished by the study investigators, who were blinded to treatment allocation. Analyses were done using SPSS (v. 26, IBM, New York).

## Results

### Feasibility

**Fig. 1:**
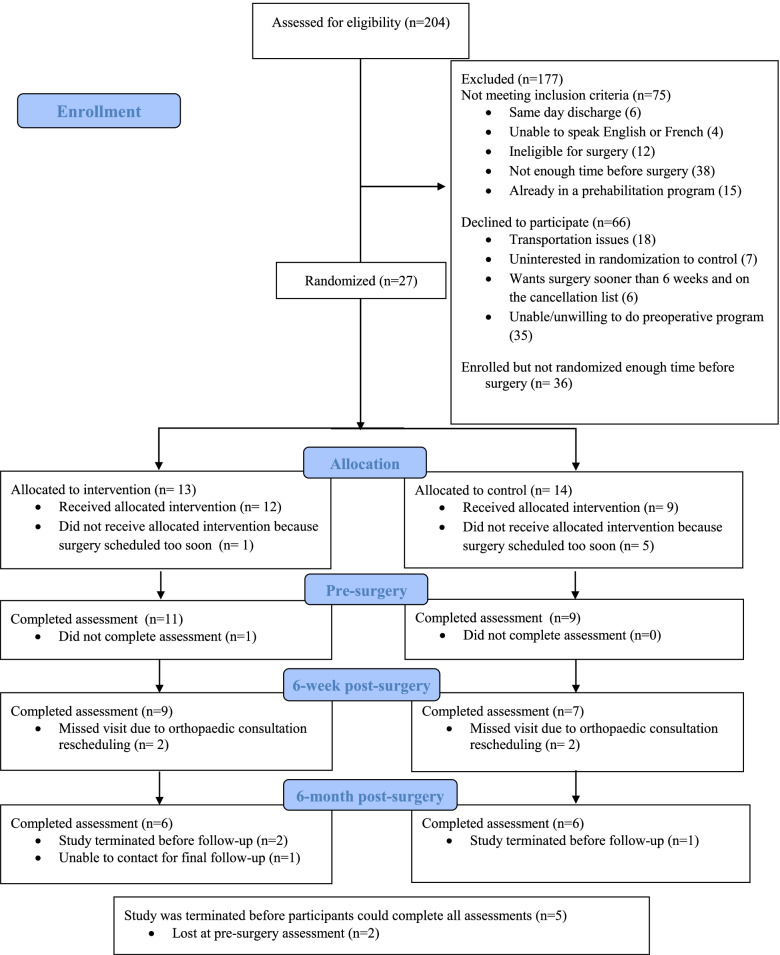
CONSORT flowchart

Of the 129 eligible participants who were approached, 63 participants consented to participate in the study (49% consent rate) (Fig. 1). The most common reason to decline participation was unable and/or unwilling to do the preoperative program. Randomization was successfully made prior to surgery for 27 participants (43% of consented participants). The other consented participants (*n* = 36) could not be randomized after baseline assessment as surgery was either not yet scheduled or when the surgery was scheduled not enough time was allocated to perform the preoperative exercise program. All 27 participants completed the baseline assessment. Patient characteristics can be found in Table 1. There was no statistical difference between the groups.

**Table 1:** Demographic of the study cohort

	Cohort	Intervention	Control	***p***-value
**Participants**	27	13	14	–
**Male:female**	15:12	6:7	9:5	0.343
**Mean age at surgery (range)**	60.6	61.1 (38.4–72.0)	60.1 (37.4–85.5)	0.497
**Mean BMI (range)**	29.2	31.5 (23.1–48.3)	27.1 (22.3–38.9)	0.120
**Hip:knee**	24:3	10:3	14:0	–

Of the 27 randomized participants, 21 (78%) did not have surgery during the exercise period, while 6 (22%) did. Of these 21 participants, 20 participants (95%) completed the pre-surgery assessment, and 16 participants (76%) completed the 6-week post-surgery assessment. The study was terminated before five participants could be eligible for the 6-month assessment. Of the remaining 16 participants, 12 (75%) completed the 6-month post-surgery assessment. The predominant reason for missed 6-week or 6-month assessments was due to orthopedic consultation rescheduling without notifying the research coordinator.

### Study challenges and protocol changes

Several changes were made to the study protocol due to challenges in reaching intended recruitment targets, successful completion of the intervention, and participant retention. First, to increase patient eligibility, patients undergoing unicondylar knee arthroplasty or hip resurfacing due to OA or other localized, non-inflammatory joint conditions were also included. These procedures were initially excluded as they are not commonly performed by the general orthopedic community. The exclusion criteria were amended to exclude patients always needing a mobility aid (walker, crutches, or wheelchair, excluding cane) to mobilize since it limits their capacity to accomplish the preoperative exercise program.

Second, the recruitment strategy was modified from administrative assistants contacting the study coordinator to the surgeons contacting the coordinator. The initial recruitment method relying on administrative assistants was not optimal as they tended to book the earliest available time in the surgical calendar, which was often within the exercise period. Since there were no common scheduling methods between assistants, this complicated the maintenance of a patient screening log. A new recruitment strategy was implemented that required surgeons and/or residents to contact the research coordinator if a patient was cleared for surgery and deemed eligible after surgeon consultation. Six surgeons received a half-hour training session by the study coordinator, covering the inclusion and exclusion criteria of the study and recruitment logistics. Orthopedic surgeons screened the incoming patients for eligibility through an admission checklist provided by the study coordinator. If eligible, the surgeon asked the patient if they would be comfortable speaking with the clinical research coordinator before leaving the orthopedic clinic. If the patient agreed to speak to the coordinator, the coordinator was notified, and they approached the patient at the end of their clinic visit. After recruitment by the research coordinator, the administrative assistant would be notified to allow the required intervention time before surgery. However, an audit demonstrated that the research coordinator had not been contacted for many patients, most probably explained by competing interests and limited time availability of surgeons and/or residents. Following this, the final method of recruiting patients was implemented, by having a research coordinator present where surgical consultations were accomplished and by querying surgeon and/or residents in real time on patient eligibility once the surgery was cleared.

Third, the burdensomeness of the intervention (exercise program duration and follow-ups) was the principal reason for declining participation in the study (*n* = 35, Fig. 1). Consequently, and as the time before surgery was also a problem, the exercise program duration was reduced from 8 to 6 weeks. To ease the data collection burden, the DEXA muscle mass assessment was removed from the protocol, as it required participants to visit a separate facility. Finally, the study visits were coordinated to combine scheduled physiotherapy visits with outcome measure visits. Nonetheless, these modifications did not have a significant impact on the recruitment rate.

## Discussion

Rehabilitation for patients undergoing lower limb JA usually includes postoperative exercises to optimize muscle strength and patient function, yet patient condition shortly after surgery is not optimal to accomplish intensive exercises needed to address deconditioning and decreased function [23, 25, 26, 28]. Alternatively, studies evaluating preoperative exercises have shown contradictory results to improve muscle deconditioning and function which can be attributable to methodological issues, exercises not specific to fundamental muscle groups, poor intervention details to allow reproduction, and outcomes measured only in the long-term. Our study was designed to overcome those limitations, assessing the feasibility of a preoperative exercise program targeting specific functional muscles for patients undergoing hip and knee JA. The known methodological limitations of previous studies in the literature were addressed in this study. This study utilized a preoperative strengthening exercise program specifically targeting quadriceps and hip abductor muscles associated with knee and hip function, respectively, rather than using a generic exercise program. Furthermore, this trial assessed patient function both 6 weeks and 6 months after hospitalization, while the majority of previous studies did not assess patient function shortly after hospitalization [23, 25, 32, 33].

Findings from this study demonstrate that this study was not feasible at our clinical site in this current form despite adjustments made to the protocol and was discontinued given the recruitment rate, randomization barriers, surgery and orthopedic consultation (re)scheduling, and loss to follow-up. Recruitment rates were below the targeted cutoff even after multiple attempts to change the recruitment strategy. As previously stated, a sample of 118 patients (59 per group) would be required to conduct a RCT with enough power to find significant changes between the groups. With our recruitment rate, reaching the required number of participants would take more than 4 years, thus was not deemed feasible for our clinical site. The consent rate was under the target even after reducing the intervention burden by decreasing the exercise program length. A low consent rate could be explained by the common belief in patients that activity and exercise can further damage the joint [61]. Loss to follow-up rates were higher than the targeted cutoff even after combining outcomes assessment with clinical encounter visits. Patients undergoing JA tend to avoid travel they deem unnecessary because of pain and limited mobility [61, 62].

### Implication for practice and future research

This study allowed for significant insight to be gained into the intricacies of the administrative and clinical flow at our clinical site, as well as an exploration of barriers to patient engagement with a preoperative exercise regimen. The results of this study cast doubt on the feasibility of an in-person preoperative rehabilitation program for patients undergoing JA in our setting. With appropriate changes, a preoperative muscle strengthening program could still be of benefit in both short- and long-term recovery after hip and knee JA.

Given the low recruitment rates due to administrative workflow, feasibility could be increased by a computer system allowing the identification of eligible patients based on clinical characteristics with information automatically relayed to research staff. Furthermore, the recruitment strategy should emphasize the benefits of exercise and reassure that exercises will not further damage the patient’s joints [61].

Another direction to improve feasibility is to adapt the preoperative rehabilitation program to a telerehabilitation program to ease the burden on the site and patients. Telerehabilitation provides the opportunity for patients to follow their rehabilitation at a distance by connecting the patient to a rehabilitation provider through video technology. The results of a meta-analysis evaluating the effect of telerehabilitation following JA show equivalent efficacy between onsite rehabilitation and telerehabilitation [63]. Furthermore, a study assessing the feasibility of a telerehabilitation program and in-person prerehabilitation program compared to usual care for JA patients found that while patients reported a high level of satisfaction towards the telerehabilitation program, there were no significant differences reported in outcome measures between the groups [64]. All outcome measures, including performance-based measures, could also be completed via telerehabilitation. However, more studies are needed to investigate feasibility and reliability.

### Limitations

Our study has limitations to consider. First, an audit of orthopedic surgeon recruitment was completed to evaluate the recruitment strategy of the pilot study, but the quantification of eligible patients missed by surgeons was not documented. Second, one participant in the intervention group did not complete the pre-surgery assessment, but the reason was not documented. The following limitations have no impact on a pilot RCT, but would be applicable in a future larger-scale RCT. Since the validity of the TUG has been established in older patients, having an age recruitment criterion of 18 years and older could be problematic if a substantial number of very young patients were recruited. However, the age distribution of the recruited sample was similar to patients typically receiving elective arthroplasty [65]. Because of the nature of the intervention, participants were not blinded to study allocation, which could influence study outcomes. Moreover, adherence to the preoperative strengthening exercise program was not assessed in this pilot study, since the intention to treat was applied.

## Conclusion

This pilot randomized controlled trial of a preoperative strengthening exercise program for patients undergoing hip or knee arthroplasty demonstrated to not be feasible in its current form at our clinical site due to the low recruitment rates and high loss to follow-up. Future investigations into a modified intervention via telerehabilitation should be explored.

## Data Availability

The data that supports the findings of this study are available from the corresponding author, SP, upon reasonable request.

## Appendix

Table 2

**Table 2:** Hip and knee preoperative exercise programs

Weeks	Hip	Knee
1	**Supine hip glide:** While the patient is lying prone with a pillow under the torso, he/she will slightly lift the operative leg and move it sideways through as much range as the hip allows. The leg will then return to the start position.	**Knee extensions supine**: While the patient is lying prone with a foam roller beneath their thigh on their operative side, the patient will lift his/her heel off of the floor as high as he/she can then return the leg to starting position.
2	**Standing hip abduction:** The patient holds on to a horizontal surface for equilibrium, such as a table or counter. The patient abducts the operative hip through as much range as the hip allows and returned to the starting position.	**Sitting knee extensions**: The patient will sit in a chair with his/her back straight. The operative knee will be straightened as much as possible then returned to starting position.
3	**Side-lying hip rotation:** The patient will lie on his/her non-operated side, with the hips straight and knees bent 90°. A pillow is placed between the knees. The hip is then externally rotated through as much range as the hip allows and returned to the starting position.	**Partial squat**: The patient will stand straight using support from a table or chair with feet shoulder width apart. The patient will perform a semi-squat then return to starting position.
4	**Side-lying hip rotation with elastic:** The same position as the week 3 exercise, but an elastic will be placed above the knees.	**Mini-squat**: The patient will stand on their operative leg, using a table or chair for balance. The patient will bend their support knee slightly then return to starting position.
5	**Leg abduction with elastic:** The same exercise as week 2, but with a yellow elastic band placed just above both ankles. The length will be determined so that elongation is at 200% at a maximum range of motion of the hip of the patient.	**Step-up**: The patient will stand with his/her operative knee on a stair. The patient will slowly shift weight onto their operative leg on the stair and step up, then step back down leading with their uninvolved leg.
6	**Side-lying abductions:** The patient will lie in the same position as weeks 3 and 4. However, their top (operative) leg will be straight. The patients will move their leg supine for ~ 6 in. then return to the starting position.	**Sit to stand**: The patients will place a chair with their backs against a wall for support. The patients will sit on the edge of the chair, fold their arms across their chest, and sit such that their knees are bent over their toes. The patients will lean forward and stand up fully, then return to starting position.

## Abbreviations

DEXA: Dual-energy X-ray
HOOS: Hip Disability and Osteoarthritis Outcome Score
JA: Joint arthroplasty
KOOS: Knee Injury and Osteoarthritis Outcome Score
LOS: Length of stay
MCID: Minimal clinically important difference
RCT: Randomized controlled trial
OA: Osteoarthritis
SD: Standard deviation
TUG: Timed-Up-and-Go
WOMAC: Western Ontario and McMaster Universities Osteoarthritis Index
